# Clinical implications of lateral head mis-centering on eye lens dose in pediatric computed tomography examinations: a phantom study

**DOI:** 10.3389/fmed.2026.1838211

**Published:** 2026-07-30

**Authors:** Mohammad Aljamal, Zuhal Y. Hamd, Awadia Gareeballah, Mousa Shaheen, Lama Almudaimeegh, Amna M. Ahmed, Mohammed Alharbi, Abbas Abuthabet, Zaid Daraghmeh

**Affiliations:** 1 Faculty of Allied Medical Sciences, Department of Medical Imaging, Arab American University, Jenin, Palestine; 2 Department of Radiological Sciences, College of Health and Rehabilitation Sciences, Princess Nourah bint Abdulrahman University, Riyadh, Saudi Arabia; 3 Department of Diagnostic Radiology, College of Applied Medical Sciences, Taibah University, Madinah, Saudi Arabia; 4 Shahin X-ray and MRI Medical Center, Hebron, Palestine; 5 Department of Internal Medicine, College of Medicine, Princess Nourah bint Abdulrahman University, Riyadh, Saudi Arabia; 6 Department of Radiological Sciences, College of Applied Medical Sciences, King Khalid University, Abha, Saudi Arabia; 7 Department of Medical Imaging, Patients Friends Society - Al-Rahma Hospital, Nablus, Palestine

**Keywords:** CT mis-centering, eye radiation dose, head phantom, lateral head shifting, pediatric brain CT

## Abstract

**Background and aim:**

Patient mis-centering during pediatric CT examinations can significantly affect radiation dose distribution, particularly to radiosensitive organs such as the eye lens. This study quantitatively evaluates the effect of lateral head mis-centering on eye lens dose and investigates discrepancies between measured and system-reported dose metrics using a low-cost pediatric head phantom.

**Materials and methods:**

A low-cost tissue-equivalent pediatric head phantom was constructed locally and validated using CT number comparisons with reference tissue values. Eye lens dose was measured using a pencil ionization chamber at the isocenter and at ±1–3 cm lateral displacements on two CT systems. Measured dose length product (DLP) values were compared with CT-reported DLP and air kerma-derived estimates. The data was analyzed statistically using Pearson correlation, paired sample t-tests, and one-way ANOVA.

**Results:**

Strong correlations were observed between isocenter and off-center doses (*r* = 0.994–0.998, *p* < 0.001). Although positional differences were not statistically significant (*p* > 0.05), a consistent asymmetric trend was observed, with higher doses on the right side. System-reported DLP substantially overestimated measured values (e.g., 720 vs. 304.83 mGy·cm).

**Conclusion:**

Lateral mis-centering introduces systematic dose distribution asymmetry and significant discrepancies in CT dose reporting. These findings highlight the importance of accurate patient positioning and caution in interpreting system-reported dose metrics in pediatric imaging.

## 1 Introduction

Concerns regarding radiation exposure in pediatric CT imaging have increased due to the heightened radiosensitivity of developing tissues and the potential for long-term effects (1). The pediatric eye lens is particularly radiosensitive to ionizing radiation (2). Therefore, high radiation doses caused by CT scans have been linked to the creation of cataracts in some cases (3). Selecting age-appropriate CT protocols, ensuring the highest level of protection and the best chance of producing a good diagnostic result (4). Several studies have found that a child is at greater risk for the damaging influences of radiation; consequently, they require cautious examination of the radiation dose given in the course of CT imaging (5).

There are two well-documented methods for evaluating the total radiation dose: dosimetry and phantom studies. These methods can be used to estimate and ultimately reduce the amount of radiation exposure for patients, particularly in pediatric brain CT scans. These techniques enable accurate radiation dose quantification and support protocol optimization, and eventually, for the safety of pediatric patients with CT examinations (6, 7). The high cost of anthropomorphic phantoms remains a major barrier to the practical use of certain technologies in radiation measurement and dosimetry. For example, the RANDO® phantom may cost tens of thousands of dollars. However, this cost limits their accessibility, particularly in low- and middle-income settings. This leads to a variation in the assessment of the quality and safety of the radiation dosage form for pediatric patients. To address this issue, quality control and dose quantification of CT can be achieved by creating substitute phantoms using inexpensive materials readily available in the local market. Such experimental data show that standard phantoms, such as the CTDI (computed tomography dose index) phantom, are not comparable to anthropomorphic phantoms in terms of dose measurement (8–10). While dosimetry techniques have been developed to improve the practice of imaging, and to aid in ensuring that the radiation dose to children is kept as low as possible. One of the components of this evaluation includes the volume of CT dose index (CTDIvol) and dose-length product (DLP), which are commonly used to measure the radiation doses received during CT examinations and assist in determining appropriate imaging techniques for the patient (11).

The eye lens dose during brain CT examinations has been reported as being wide in previous studies with doses of 10.5 to 34.2 mGy (12). One phantom study with a 10-year-old phantom found a dose of 28.11 mGy on the eye lens for brain CT (13). Another study has noted the effect of repeated imaging, with an average total dose to the eye lens that could reach 168 mGy (14). Likewise, a study has identified the significant differences in eye radiation doses received with brain CT scans ranging from 2-130 mGy (3). Depending on the CT examination protocol, eye lens doses have also been reported within the ranges of 25.72 ± 6.12 mGy and 49.07 ± 10.08 mGy (15). However, the radiation dose from a single brain CT scan is typically less than the dose that is known to cause cataract formation, but repeated scans may increase the dose to the recommended threshold of 0.5 Gy (16). Therefore, frequent radiation dose evaluation is crucial due to this variation in radiation dose values. Some studies have suggested the use of dose optimization methods such as gantry tilt, organ-based tube current modulation, lower tube current, and using shielding to reduce radiation dose to the eye lens (17, 18).

From another perspective, the wide variation in brain size, which is influenced by age and physical development, particularly in young children, presents challenges for some CT technologists (19). Particularly, placing the child properly in the CT scanner to access the precise isocenter of the brain. Consequently, the radiation dose that patients receive from brain CT scans will be affected (20). Where centering accuracy is essential in order to reduce the amount of unnecessary radiation. Vertical off-centering may lead to significant changes in the organ doses that may reach 34 and 19%, in the infratentorial brain and the eyes, respectively (21). Previous studies had indicated that CTDIvol increases significantly when it is moved vertically off-center as little as several centimeters based on measurement using standard cylindrical polymethyl methacrylate (PMMA) (20). Another study showed that dose distribution in the head can vary depending on patient setup errors and head size (4). This variation is influenced by the distance between the X-ray tube and the beam section width (22). While previous studies have primarily focused on vertical off-centering and generalized dose variations, limited attention has been given to lateral mis-centering and its specific impact on eye lens dose in pediatric populations. Moreover, anthropomorphic phantoms are not easily obtained in most low-income countries because they are expensive. Thus, this paper presents a low-cost, tissue-equivalent pediatric head phantom and a systematic quantitative analysis of the dose measured by the phantom and the air kerma estimates versus the DLP reported by the CT system. These two approaches, focusing on lateral mis-centering and dose reporting accuracy, are a practical and clinically significant addition to the optimization of pediatric CT dose.

## 2 Materials and methods

### 2.1 Fabrication of a pediatric head phantom

A new tissue-equivalent material was proposed in this study, which is based on olive oil. The preparation was carried out using 50% olive oil, 20% sodium hydroxide, and 30% water. First, the olive oil was placed in a metal container and heated to a high temperature (~150–200 °C) for thirty minutes. The high heat ensures that sodium hydroxide has reacted with the olive oil, lowering the viscosity of the oil. This process ensured a homogeneous mixture and removal of air bubbles. The mixture was then constantly mixed while the remaining components were added and allowed to set overnight. Three samples were prepared to assess the CT number compatibility with the value of the average CT number of grey and white matter of the brain tissue (23). A bone sample was also fabricated based on a previous study using 74% distilled water and 26% calcium sulfate (24). The samples were scanned using a Philips Ingenuity 128-slice CT (Philips, Netherlands). The scanning protocol was informed by the standard protocol that is normally used to scan the brain of a child (100 kV, 250 mAs, and 3 mm slice thickness). An ROI of 20 pixels in the shape of a circle was placed on the CT image of the respective material to determine the CT number. Each material’s CT results faithfully captured the density properties of human tissue. The fabricated phantom materials were validated by comparing measured CT numbers (Hounsfield Units) with reference values reported in the literature for pediatric brain tissue and bone. The obtained ranges (20–40 HU for soft tissue and 400–1,000 HU for bone) were consistent with published data, supporting the suitability of the phantom for dosimetric assessment (25). To determine representative pediatric head dimensions for phantom fabrication, a retrospective analysis of 124 brain CT examinations of patients aged 2–16 years was conducted using a Picture Archiving and Communication System (PACS) between May 2024 and March 2025. Patients outside this age range, particularly those less than two years old, were excluded due to significant variations in developmental anatomy and head size (26). The exclusion of older pediatric patients (11–16 years) is due to their distinct anatomical characteristics and the use of different CT protocols. Therefore, the head dimensions of 97 pediatric patients were measured manually in three orthogonal planes—axial, coronal, and sagittal—to ensure accuracy and consistency. At least three measurements per dimension were obtained and averaged to minimize measurement variability. These measurements were used to determine the anteroposterior (AP) length, right–left (RL) width, and cranio-caudal height of the head. Since the patients were within the 3–10 year age range; therefore, this group was selected as the target population for phantom design. The resulting mean dimensions were 13.3 ± 1.3 cm (AP cranium length) and 18.7 ± 1.6 cm (AP length from the mental protuberance of the mandible to the top of the skull vertex), 12.8 ± 1.1 cm (RL width), and 15.4 ± 1.5 cm (height), ensuring anatomical relevance of the fabricated phantom to the selected pediatric population.

After evaluating the CT number of each material and determining the size of the pediatric head, the bony part of the head was prepared based on the concentration examined in the previous step. Then it was cast in a silicon mold that was made to the size of a child’s brain, as it was identified before in the CT images. A large amount (2 liters) of olive oil mixture was then prepared in the same manner and poured into the prepared skull. A hole was drilled to accommodate a pencil CT chamber (Raysafe X2 dosimeter, Sweden), allowing the dosimeter to be placed exactly in the center of the phantom’s eye. The phantom validation aimed to define radiological attenuation similarity enough to enable a comparative dosimetric evaluation under controlled experimental conditions. While absolute dosimetric equivalence cannot be achieved by Hounsfield Unit matching alone, this technique does show that the attenuation properties of the fabricated materials are similar to those of the pediatric soft tissue and bone during CT exposure. The phantom was suitable for the study to assess relative dose variation due to positional mis-centering as opposed to absolute organ dose estimation, as this was the main aim of this study. The CT images of the pediatric patients were analysed to assess the protocol commonly used in two types of CT, namely, the Philips CT scanner 7,500 Spectral CT machine (Philips, The Netherlands) and the Philips Ingenuity 128-slice CT scanner machine (Philips, The Netherlands). It was determined that the CT technologists utilize various imaging parameters on the different CT systems, as shown in Table 1.

**Table 1:** The scanning parameters that are usually utilized in brain imaging of pediatric patients in two different CT scan systems.

	Spectral CT 7500		CT Philips-128 slice		
Parameters	# 1	# 2	# 3	# 1	# 2
Tube voltage (kV)	120	120	120	100	120
Tube current (mAs)	250	250	320	180	350
Slice thickness (mm)	2	2	2	3	3
Pitch	0.29	0.4	0.4	0.7	0.4
Rotation time (sec.)	0.33	0.4	0.5	0.4	0.4

**Figure 1:**
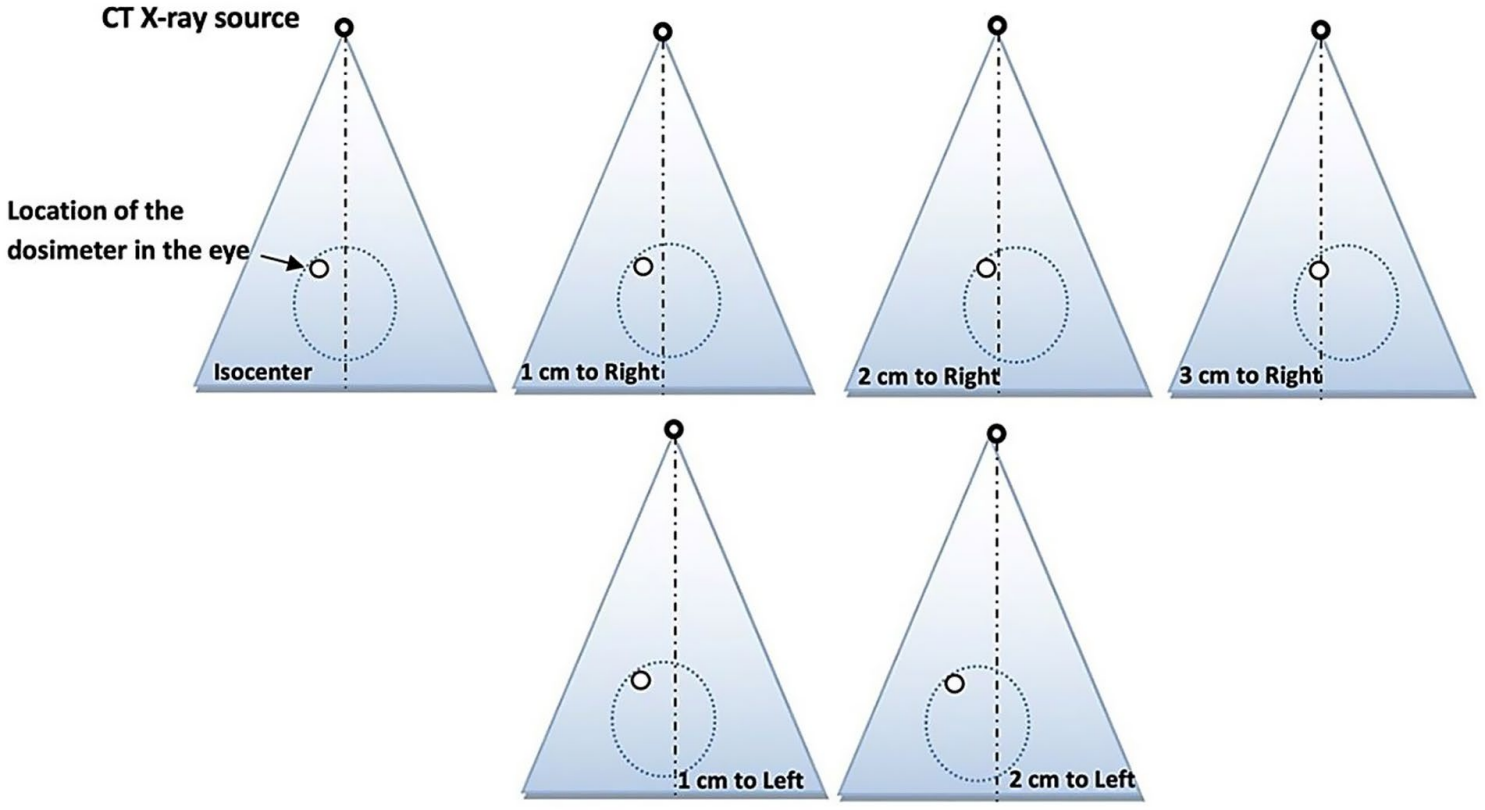
An illustration of the expected transverse shifting in the position of the patient’s head, 1–3 cm to the right and 1–2 cm to the left of the isocenter.

**Figure 2:**
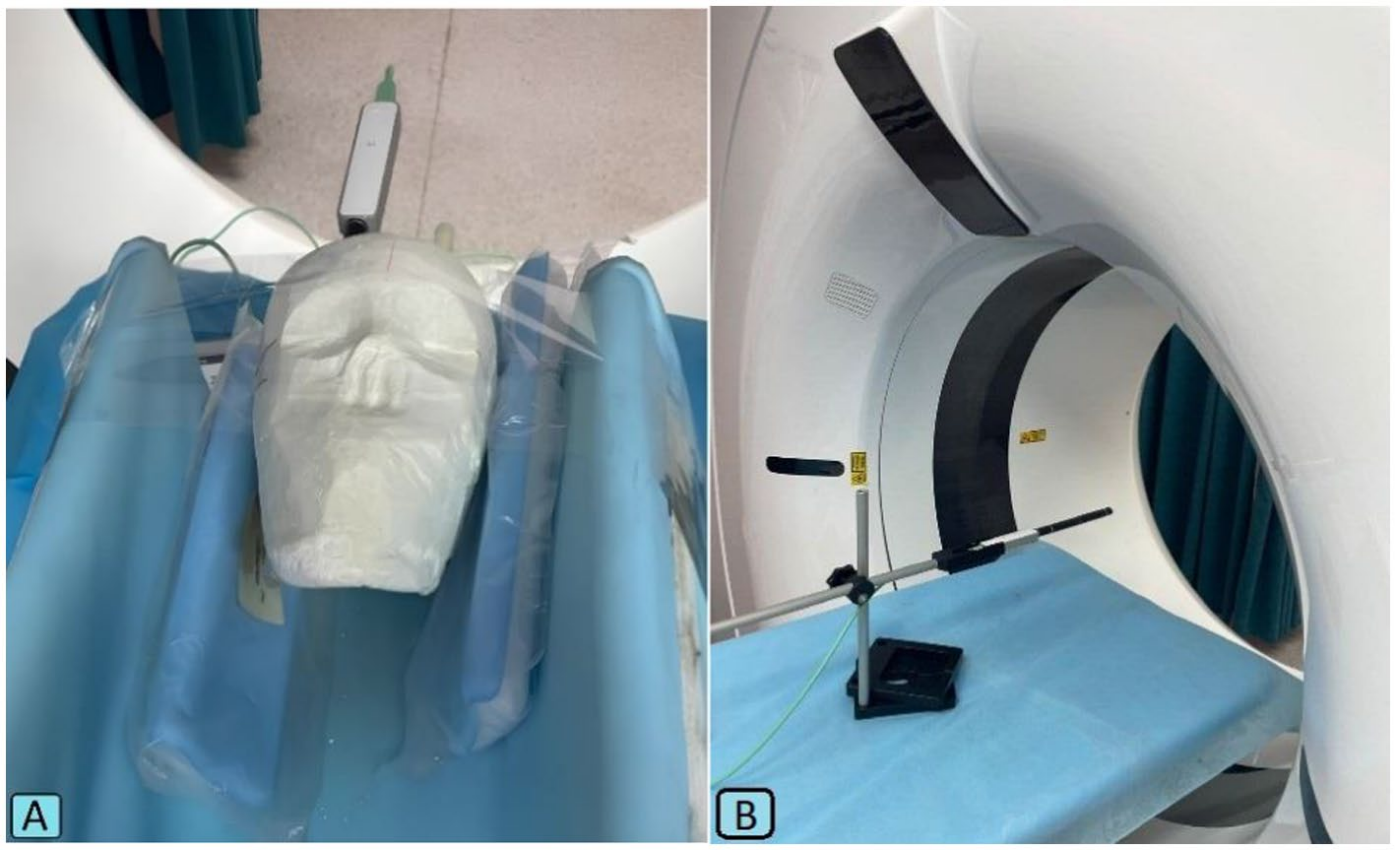
**(A)** Fabricated brain phantom during a CT scan, after inserting a CT pencil ionizing chamber in the middle of the right eye. **(B)** Pencil CT chamber location during the air kerma index measurement at the center of the beam for the selected protocols.

Since the primary objective of the study is to assess the lateral shifting of head-on eye radiation dose, the phantom was positioned on the CT table and aligned with the gantry isocenter using standard laser guidance, after which lateral displacement was introduced incrementally in 1 cm steps up to ±3 cm along the X-axis to the right and ±2 to the left. A schematic diagram of the experimental setup has been added (Figure 1) after placing the dosimeter at the anatomical location corresponding to the center of the right eye (Figure 2A). The first measurement was done by placing the pencil ionization chamber at the anatomical center of the right eye inside the phantom in the fabricated phantom. The DLP was measured by multiplying the CTDIvol value obtained at the center of the eye level by 3.2 cm (the superior–inferior opening length of the orbit) using the following equation:

DLP (mGy·cm) = CTDIvol (mGy) × Irradiated Scan Length (cm).

The CTDIvol measurements were also conducted in the frontal bone region and the maxillary region, which were adjacent to the anatomical parts and the attenuation characteristics of the CT examination. The position of the ionization chamber was set at the center of the frontal bone, and the value of the CTDIvol was multiplied by 7 cm, which is similar to the average length of a cranial scan above the eyes. In addition, the CTDIvol in the maxillary region was multiplied by 8.5 cm, which is the distance between the inferior orbital margin and the mental protuberance of the mandible. Finally, the three DLP values obtained were added up to estimate the average total DLP contribution to the eye region during the entire brain examination (CT). For comparison purposes, the air kerma index was also evaluated at the center of the beam for the selected protocols, as shown in Figure 2B. The same method and anatomical length distribution were used to calculate the air kerma to compare the DLP value between the phantom and the air kerma-based estimate. The measurements were repeated three times for each protocol of CT scan to ensure reproducibility of measurements and ensure accuracy of measurements. These measurements were not made for absolute dosimetry using CTDI, but were taken to compare dose variance due to lateral mis-centering. The length of the scan was matched with the clinically applied brain CT acquisition length for each protocol.

The DLP value derived from a CT scan was also evaluated, which is based on measurements in a Polymethyl methacrylate (PMMA) head phantom. The DLP for each protocol of both CT scans was compared to assess the difference in radiation dose. Statistical analysis was performed using SPSS version 27 (IBM, Armonk, NY, USA), Jamovi software version 2.6 (Sydney, Australia), and numiqo online Statistics Calculator (numiqo Team, Austria). As the measurements of doses yield a normal distribution in the isocenter, and with shifting to the right and left (Shapiro test *p* values >0.05), a paired sample t-test was used to assess the relationship between isocenter and off-center dose measurements, and one-way ANOVA was applied to evaluate differences among measurement methods (phantom, air kerma, and CT-reported DLP). Furthermore, a correlation heatmap Pearson correlation coefficient was used to assess the impact of exposure factors (kVp, mAs, pitch, and rotation times) on DLP measurements on the phantom and to assess the correlation between the DLP value in the isocenter compared to the variables shift obtained.

## 3 Results

**Figure 3:**
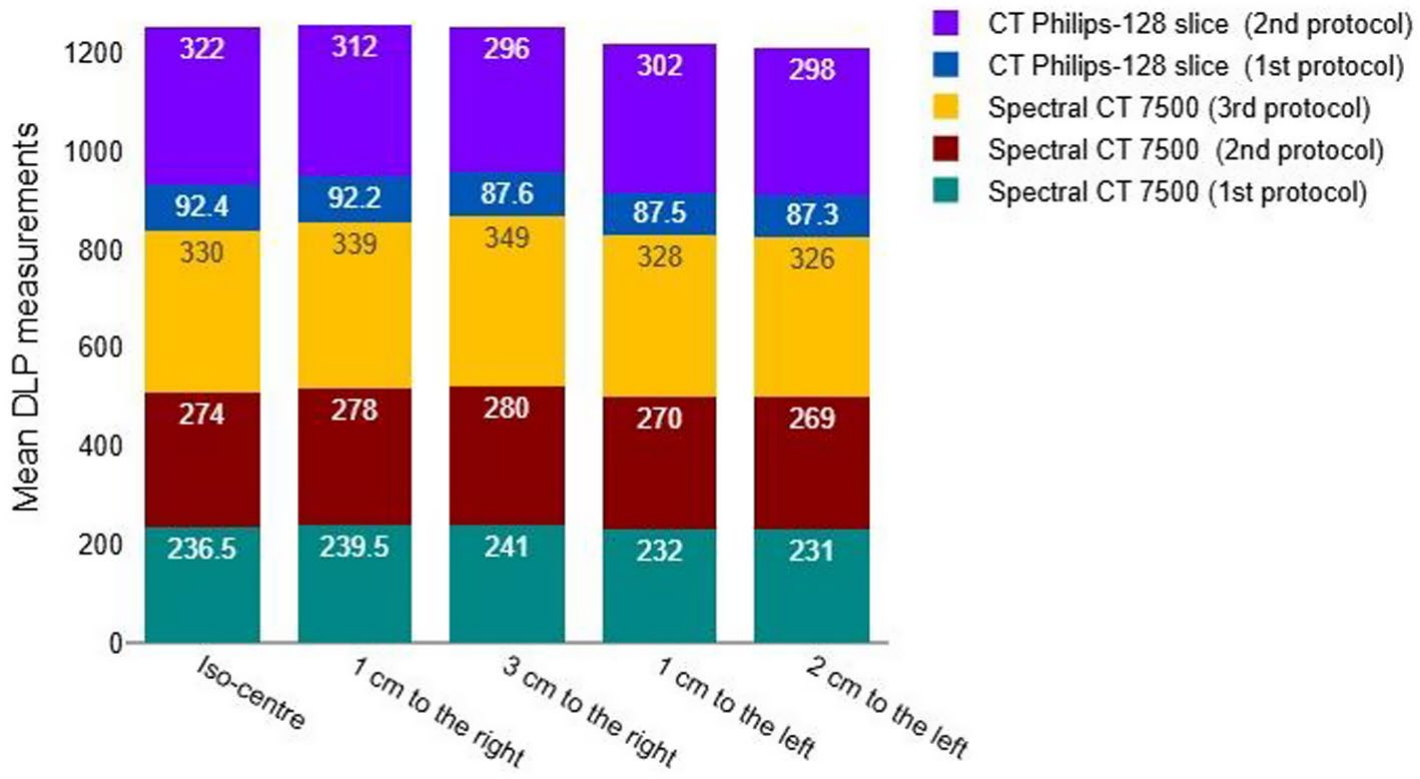
Illustrate the relationship between lateral displacement and measured DLP (mGy·cm).

Measured dose-length product (DLP) values in the center of the eye of the fabricated pediatric head phantom for all protocols for lateral shifting positions are detailed in Table 2. Correlation and paired difference were conducted to measure the dose distribution. A strong positive correlation was observed between the isocenter dose and dose at all peripheral positions (right and left). Figure 3 illustrates the relationship between lateral displacement and measured DLP, demonstrating a gradual and asymmetric trend in dose variation. This trend, although not statistically significant, suggests a consistent directional influence of lateral mis-centering. Table 3 indicates that the Pearson correlation coefficient (r) was remarkably high, with the value ranging between 0.994 and 0.998, and that all the correlations were statistically significant (*p* < 0.001). These results indicate a highly consistent and predictable dose distribution.

**Table 2:** Measured dose-length product (DLP, mGy·cm) in the eye at the isocenter and peripheral positions for different CT systems and scanning protocols.

	DLP (mGy·cm)					
Type of system	Iso-center	1 cm to the right	2 cm to the Right	3 cm to the right	1 cm to the left of the iso-center	2 cm to the left of the iso-center
1st protocol (Spectral CT)	236.5 ± 0.1	239.5 ± 0.01	240 ± 0.04	241 ± 0.01	232 ± 0.03	231 ± 0.1
2nd protocol (Spectral CT)	274 ± 0.09	278 ± 0.02	279 ± 0.001	280 ± 0.1	270 ± 0.02	269 ± 0.01
3rd protocol (Spectral CT)	330 ± 0.05	339 ± 0.03	344 ± 0.04	349 ± 0.03	328 ± 0.1	326 ± 0.06
1^st^ protocol (Philips-128)	92.4 ± 0.04	92.2 ± 0.07	89.9 ± 0.04	87.6 ± 0.06	87.5 ± 0.04	87.3 ± 0.1
2^nd^ protocol (Philips-128)	322 ± 0.01	312 ± 0.02	299 ± 0.1	296 ± 0.001	302 ± 0.1	298 ± 0.06

**Table 3:** Correlation analysis between isocenter and peripheral DLP measurements.

Peripheral position	Mean ± SD (mGy·cm)	Correlation coefficient (r)	*p*-value
Isocenter vs. 1 cm right	399.11 ± 195.76	1.000	<0.001
Isocenter vs. 2 cm right	398.48 ± 196.33	0.999	<0.001
Isocenter vs. 3 cm right	398.60 ± 196.51	0.999	<0.001
Isocenter vs. 1 cm left	396.16 ± 197.82	1.000	<0.001
Isocenter vs. 2 cm left	395.58 ± 198.16	1.000	<0.001

The difference in dosage between the isocenter and periphery sites was not statistically significant, despite a substantial association. Table 4 provides a summary of the paired t-test results. The *p*-values of all five peripheral conditions exceeded 0.05, which implied that the variations that were observed were within the range of the random variations in measurements. A consistent pattern was observed across all measurements at the isocenter and the periphery sites. The difference was high between DLP measurement in the isocenter, related to the left 1 to 2 cm from the isocenter, but statistically insignificant. An asymmetric dose distribution favoring the lower left dose was suggested by (t = 1.76, 1.82, and a mean difference of 2.52 and 3.11 mGy·cm, respectively).

**Table 4:** Statistical analysis of dose differences between the isocenter and peripheral positions.

Measurement position	Mean difference (DLP)	95% confidence interval	*p*-value
Isocenter vs. 1 cm right	−0.41	−2.70 to 1.87	0.702
Isocenter vs. 2 cm right	0.21	−4.22 to 4.65	0.919
Isocenter vs. 3 cm right	0.09	−5.26 to 5.44	0.971
Isocenter vs. 1 cm left	2.53	−0.57 to 5.63	0.102
Isocenter vs. 2 cm left	3.11	−0.60 to 6.83	0.093

The substantial difference between the DLP values calculated using alternative techniques and those acquired by direct phantom measurement was a crucial discovery of this investigation. A repeated measures analysis of variance for both CT systems showed statistically significant differences (*p* < 0.001) between the DLP exhibited by the CT system, the DLP computed by air kerma, and the phantom-based DLP. The Philips-128 slice system also showed this pattern (Table 5). The difference was most noticeable in the second protocol, where the CT system showed a value of 720.00 mGy·cm, whereas the phantom-measured DLP was 304.83 mGy·cm. This implies that the internalized dose estimation algorithms on both systems are likely to overestimate the actual patient dose in this specific scanning scenario and may not appropriately give the amount absorbed by a phantom.

**Table 5:** Comparison of DLP values from phantom measurement, air kerma calculation, and CT system display for the Philips-128 slice system.

Protocol	Phantom DLP Mean ± SD	Air kerma DLP	CT displayed DLP	*p*-value
Protocol 1	89.48 ± 2.38	157.80	232	<0.001
Protocol 2	304.83 ± 10.13	665	720	<0.001
Total	197.16 ± 112.68	411.40 ± 264.88	476 ± 254.85	<0.001

**Figure 4:**
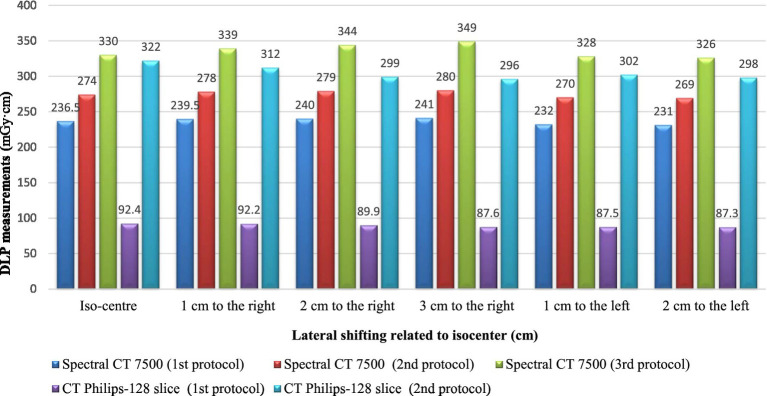
Comparison of mean phantom-based DLP values for different scan areas across two CT systems and multiple protocols.

**Figure 5:**
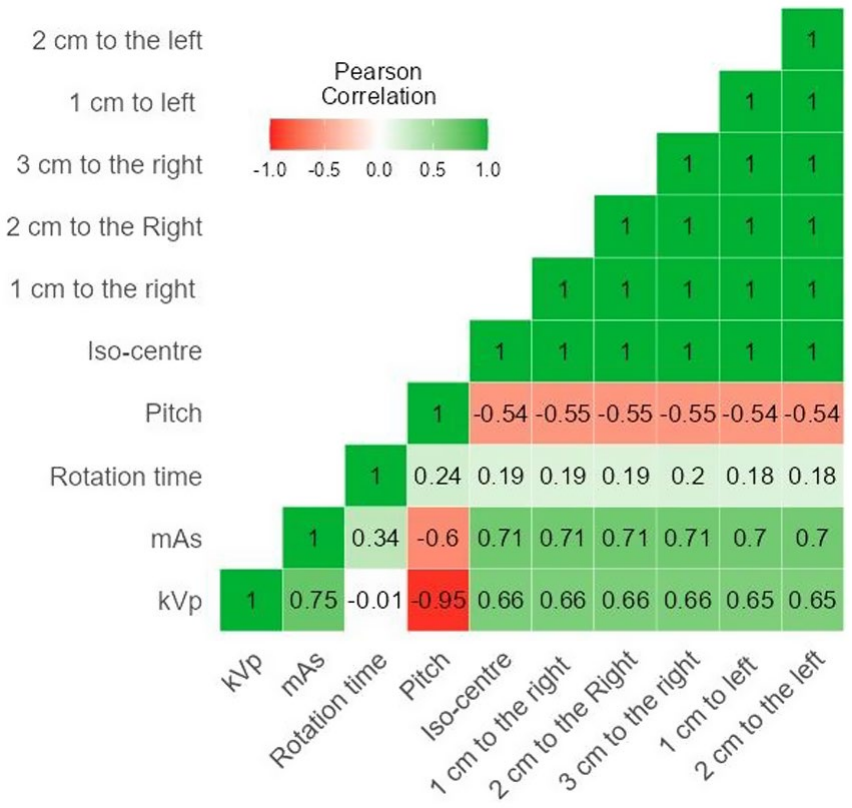
Correlation heatmap to assess the relationship between DLP measurements and kVp, mAs, rotation time, and pitch.

Phantom-measured DLP was underestimated in the system-reported DLP of the Spectral CT 7500 (Table 6). In protocol 1, for instance, the phantom-measured mean DLP was 236.67 mGy·cm, whereas the system-reported mean was 441.00 mGy·cm. Similarly, DLP based on air kerma was overestimated. The total radiation dose output was significantly different depending on the CT system and the chosen scanning protocol. Figure 3 displays a visual summary and shows that Spectral CT 7500 typically uses higher levels of absolute dose in its protocols using pediatric patients than Philips-128 slice. Furthermore, a clear and intended step-wise increase in DLP was observed with the progression of protocols within each system. For protocol 1, the average dose-length product (DLP) measured from a phantom for the Spectral CT 7500 was 236.67 mGy·cm; for protocol 2, it was 275.00 mGy·cm; and for protocol 3, it was 336.00 mGy·cm. In addition, the dose for the Philips-128 system increased significantly from 89.48 mGy·cm with protocol 1 to 304.83 mGy·cm with protocol 2. These data reveal that a protocol choice has a direct and important effect on the final dose to patients, and protocol optimization is necessary. Also, some protocols, particularly those with higher tube current settings, may reflect adult-based imaging practices rather than optimized pediatric protocols. The correlation heatmap in Figure 4 shows a strong and significant correlation between kVp, mAs settings, and DLP measurements across different shifts of phantoms, while a weak correlation of different DLPs measured with rotation time, and a negative correlation of DLP values with pitch value (Figure 5).

**Table 6:** Comparison of DLP values from phantom measurement, air kerma calculation, and CT system display for the spectral CT 7500.

Protocol	Phantom DLP Mean ± SD	Air kerma DLP	CT displayed DLP	*p*-value
Protocol 1	236.67 ± 4.29	428.90	441	<0.001
Protocol 2	275.00 ± 4.73	508	525	<0.001
Protocol 3	336.00 ± 9.40	648.90	649.10	<0.001
Total	282.56 ± 42.54	528.60 ± 93.63	538.37 ± 87.96	<0.001

## 4 Discussion

Lateral head displacement significantly influences the radiation dose distribution of the scanned child. Children’s heads are relatively small, resulting in a higher likelihood of the center of the CT scanner not being aligned in the CT bore, which results in a higher radiation dose to the child’s eye lens. Moreover, if the protocol used to scan a child was not selected properly, the child will receive a significantly higher radiation dose, particularly if the adult protocol is used on a child. These factors contribute to increased radiation exposure.

This study characterized a radiation dose delivery in-house developed pediatric head phantom during CT brain examination, with attention to the effect of the lateral shift of the head when placed in a CT scanner isocenter. Also, evaluate the differences in radiation dose between the dose measured by the head-phantom and that obtained by air kerma, and between those available for the CT scan. The main results are that the isocenter doses and the peripheral doses are strongly correlated in contrast to a very predictable manner, small yet significant directional dose distribution trends exist, and, i.e., for measured vs. estimates by CT systems, there is an inherent bias.

We found that radiation dose at the isocentre illustrated a very positive relationship (*r* = 0.99–1, *p* < 0.001) with all peripheral positions measured. This finding is important, as it establishes that the isocenter dose is a strong predictor of the dose delivered to neighboring tissues. As emphasized by Inoue et al. (4), management of radiation dose is required to optimize imaging protocols and to detect and prevent examinations with excessive radiation exposure, and a predictable relationship between central and peripheral doses is crucial for this effort. Correlation, however, does not mean the same dose values. Although the absolute differences were not statistically significant in our paired analysis, which showed a general homogeneity of the doses across the phantom, there was a general directional movement. The doses recorded to the left of the isocenter were consistently and significantly lower than the doses measured to the right, which were slightly higher. This is consistent with previous phantom studies that have found that, unlike vertical off-centering, lateral shifts do not significantly change dose measures, mainly because of the rotational nature of CT acquisition and the comparative symmetry of the attenuation profile of the head. For example, Isa et al. (20) observed significant changes in dose per organ with vertical mis-centering compared to lateral displacement, with CTDIvol values increasing significantly when the phantom was displaced, and Euler et al. (21) found that vertical mis-centering caused a greater change in dose per organ than lateral displacement. Notably, a recent study by Ravenscroft et al. (27) further confirmed that radiation dose was not affected under lateral conditions, and that significant dose changes occurred with vertical displacement, with the effects of automatic tube current modulation. A previous study has shown significant spatial dose variations, including directional differences within the same plane, particularly under off-centering conditions and due to the effect of bowtie filters and beam modulation mechanisms (28). The bowtie filter intentionally creates a non-uniform beam intensity profile, with higher intensity centrally and lower intensity peripherally. The optimal functioning of a bowtie filter assumes the patient is centered on the scanning isocenter, but off-center positioning, particularly vertical position, can alter the radiation dose and image quality (16). Therefore, when the object is not perfectly centered at the isocenter, this modulation can lead to asymmetric dose distribution, resulting in higher doses on one side and lower doses on the other. Collectively, these results confirm that errors in small lateral positioning are the primary contributors to dose distribution but not absolute dose, which, nonetheless, may still introduce clinically significant asymmetry in radiosensitive organs, such as the eye lens. In pediatric imaging, even small increases in radiation dose to radiosensitive organs may contribute cumulatively to deterministic effects, including cataract formation. Reported eye lens doses in this study fall within ranges documented in previous literature; however, cumulative exposure from repeated CT examinations may approach the threshold for radiation-induced cataract formation (~0.5 Gy). This highlights the importance of minimizing unnecessary exposure through proper positioning and protocol optimization.

A key finding of this study is that our research showed a huge difference between the DLP values obtained through direct fabricated phantom measurement and estimated through the built algorithms of the CT systems. In both the Spectral CT and Philips-128 systems, the DLP reported by the console was, on average, significantly larger than the DLP measured on the phantom. This has direct implications for clinical dose optimization and quality assurance practices, which frequently depend on these system-reported values. This is consistent with the findings of Inoue et al. (4), who emphasized the importance of accurate dose estimation methods. When clinicians and medical physicists use inflated DLP values as the basis for protocol modifications, they may be misled into thinking that a patient dose is greater than it actually is, which may result in additional limitations or an erroneous risk–benefit analysis (4). This finding is consistent with previous reports on CT dose estimation limitations, which have been brought out in several dosimetry studies. Although the phantom used in this study was not validated against a commercial anthropomorphic phantom or independent dosimetry systems such as thermoluminescent dosimeters (TLDs), its design incorporates key anatomical and radiological features relevant to pediatric head imaging. The measured CT number ranges for soft tissue (20–40 HU) and bone (400–1,000 HU) are consistent with reported human tissue values, supporting radiological equivalence (25). The discrepancy between system-reported and measured DLP values is likely attributable to the use of standardized PMMA phantoms, which are homogeneous and lack anatomical detail in CT dose calculations, which do not account for anatomical heterogeneity, particularly the higher attenuation properties of bone. Bone tissue has significantly higher CT numbers than soft tissue, reflecting major differences in their physical composition. While soft tissues such as muscle typically fall within a narrow range of about 50 to slightly above 80 HU and show little dependence on scanning energy, bone exhibits a much wider and higher range, from roughly 458 up to over 2000 HU, depending on density and scan settings, which leads to stronger X-ray attenuation. In contrast, soft tissue is closer to water in composition, resulting in lower and more stable attenuation, which explains the relatively small variation in its CT numbers (29). The present phantom is more representative in terms of anatomical structure to assess organ-specific dose variation, especially for the eye lens. The use of the controlled model of a single phantom throughout the study guarantees a high degree of reproducibility and internal consistency under all experimental conditions, even though anthropomorphic phantoms are the standard for absolute dosimetry. Thus, the phantom is acceptable for relative dose comparisons and evaluation of the positional effect effects, which are the main goals of this study, but not as an absolute dosimeter. The observed overestimation is not solely due to the difference in tissue attenuation. In addition, it assumes a certain beam geometry, scatter condition, and bowtie filter performance when using scanner-specific dose estimation algorithms. These factors, combined with variations in patient positioning, may lead to systematic discrepancies between reported and actual absorbed dose. This results in systematic overestimation when applied to anatomically realistic conditions. Therefore, clinical practice will be significantly impacted by this overestimation.

In terms of clinical practice, international guidelines like the International Commission on Radiological Protection (ICRP) and International Atomic Energy Agency (IAEA) have stressed the importance of accurate patient positioning at the gantry isocenter as an important point for dose optimization, but do not specify numerical limits for acceptable mis-centering. Practical quantitative guidance is proposed, based on the results of the present study. For gaps of up to ±1 cm from the isocenter, the dose variation was below ~2%, which can be considered as optimal positioning. Displacements of ±1–2 cm can create a slight, but detectable asymmetry, and this should be avoided if possible. If the displacement is greater than ±2 cm, there is a clear directional dose change, and this type of positioning should be regarded as suboptimal. Overall, dose differences of up to ~5% were seen at ±3 cm, but while small for a single scan, this may be clinically significant in paediatric subjects who undergo multiple CT scans.

Our study, through careful dose mapping, offers a paradigm of the exposure of lenses in usual clinical cases of patient motion or less-than-ideal positioning. The literature supports the necessity of such vigilance without any doubt. Tarkiainen et al. (17) established that using specific training, radiographers were able to make the rate of successful lens exclusion rise to 11.8% instead of 1.8%, and the modelled dose of the lens was reduced by a significant margin, to 1.5 mGy. Similar to this, Abay et al. (30) showed that lens exclusion rates may be raised from 0 to 14.5% through a quality improvement project. Together with our own research, these results show a significant discrepancy between ideal practice and actual use.

Furthermore, the importance of protocol optimization for children is crucial. LaQuaglia et al. (31) found that children undergoing head CTs in adult-focused hospitals received significantly higher radiation doses (higher DLP and CTDIvol) than those in pediatric specialty hospitals. A recent study highlighted that one of the main reasons for increasing the radiation dose of pediatric patients during brain CT scans is attributed to inappropriate practices by radiographers using adult CT protocols for pediatric patients (32). The use of non-optimized or adult-based protocols in pediatric imaging represents a significant concern for radiation protection. Inappropriate selection of scanning parameters, such as higher tube current (mAs) and tube voltage (kV), can directly increase radiation exposure to radiosensitive organs, particularly the eye lens. This issue, in combination with positioning errors, as demonstrated in this study, where lateral mis-centering introduces additional dose asymmetry and potential overexposure to one side of the head. These findings emphasize that both protocol optimization and accurate patient positioning are critical components of dose reduction strategies. Therefore, strict adherence to pediatric-specific protocols, combined with continuous radiographer training and quality assurance programs, is essential to minimize unnecessary radiation exposure and ensure compliance with the ALARA principle.

The creation of a specially designed, tissue-equivalent pediatric head phantom that precisely mimics the size and density of a real child’s head is one of this study’s key strengths. This offers a realistic and regulated dose measurement model. However, we acknowledge some limitations; the use of only a simulated pediatric head phantom does not provide the variability in anatomy found in the entire population, including those less than two years old or older than 10 years old. Because of the limited number of studies on this issue, only lateral head shifting in pediatric brain CT examinations was evaluated in this article; previous articles focused solely on vertical off-centering, which is a cause of dose variation. Additionally, a key limitation is that dose measurements were obtained from a single eye location only. As a result, lateral asymmetry was inferred indirectly rather than measured simultaneously in both eyes. Also, this study utilized two CT systems from a single manufacturer (Philips). Given differences in dose calculation algorithms between various vendor systems and system design, the findings might not be directly applicable to other vendor systems. The study did not involve any calculation of the effect of bowtie filters in reducing radiation dose measurements or any computational simulation (Monte Carlo simulation), etc. In addition, the phantom validation was based mainly on radiological tissue equivalence, which is based on the CT attenuation properties of the phantom, and not directly against a reference anthropomorphic dosimetry phantom or against organ-specific absorbed dose measurements or an independent dosimetry system like thermoluminescent dosimeters (TLDs). Hence, these findings must be considered largely in the context of comparative positional dose analysis. To improve the findings’ generalizability, future studies should use a greater variety of CT scanners and methodologies.

## 5 Conclusion

This phantom study illustrates the predictable trend in radiation dose distribution, which occurs due to head motion during CT scans in pediatric patients, and suggests the most significant finding that the CT systems themselves over-dosed the phantom. We have determined that patient placement care, strict dose surveillance, and verified dose surveillance are good practices and mandatory components of a safe pediatric neuro-imaging procedure when coupled with the vast literature data. In a clinical context, the patient positioning within the scanner isocenter ±1 cm is recommended to reduce dose asymmetry. However, any lateral displacement greater than ±2 cm is undesirable, and a displacement near ±3 cm could result in clinically meaningful dose variation, especially in the case of repeated examinations in children. Positioning aids, laser alignment verification, and regular training of the technologists are key to maintaining accurate positioning of the patient. The effective implementation of the ALARA principle in children’s CT imaging depends on such measures.

## Data Availability

The original contributions presented in the study are included in the article/supplementary material, further inquiries can be directed to the corresponding author/s.
